# First quantitative evidence of territorial behavior in squid near spawning substrates

**DOI:** 10.1038/s41598-025-14308-1

**Published:** 2025-08-08

**Authors:** Shun Tokioka, Jun Yamamoto, John R. Bower, Hajime Matsui, Satoshi Suzuki, Yasunori Sakurai

**Affiliations:** 1 Shiogama Field Station, Fisheries Resources Institute, Japan Fisheries Research and Education Agency, 3-27-5 Shinhama, Shiogama, 985- 0001 Miyagi Japan; 2 Field Science Center for Northern Biosphere, Hokkaido University, 3-1-1 Minato, Hakodate, Hokkaido 041-8611 Japan; 3 Faculty of Fisheries Sciences, Hokkaido University, 3-1-1 Minato, Hakodate, 041-8611 Hokkaido Japan; 4 Yokohama Field Station, Fisheries Resources Institute, Japan Fisheries Research and Education Agency, 2-12-4 Fukuura, Kanazawa, Yokohama, 236-8648 Kanagawa Japan; 5 Shizuoka Prefectural Research Institute of Fishery and Ocean, Iwashigashima, Yaizu, 136-24, 425-0032 Shizuoka Japan; 6 Hakodate Cephalopod Research Center, Hakodate Research Center for Fisheries and Oceans, 20-5 Benten , Hakodate, 040-0051 Hokkaido Japan

**Keywords:** Territoriality, Reproductive tactics, Spear squid, *Heterololigo bleekeri*, Behavioural ecology, Ecology

## Abstract

In loliginid squids, solitary large males have been observed near the spawning grounds in several species, but little is known about their reproductive behavior. Here we describe the behavior of spear squid *Heterololigo bleekeri* in a 300 m^3^ tank near spawning substrates. Observations were recorded on their proximity to the spawning substrates, agonistic behavior, and mating behavior. In both of two rearing experiments, individual males were observed to remain near the spawning substrates and display more frequent agonistic behavior than the other males, suggesting they formed territories near the substrates. The territorial males did not monopolize the females themselves or mating opportunities, suggesting that the benefits of territorial tactics are related to the complex fertilization patterns of this species. To our knowledge, this is the first report of territorial behavior in a squid using quantitative data.

**Supplementary Information:**

The online version contains supplementary material available at 10.1038/s41598-025-14308-1.

## Introduction

A territory is a fixed area where an animal maintains exclusive or preferential access to resources by actively excluding competitors^1^. In many taxa, male territorial behavior is associated with reproductive success^1,2^. Territorial males increase their reproductive success by defending reproductive resources such as mates or spawning sites, primarily by controlling access to the resources. For example, in northern elephant seals *Mirounga angustirostris*, territorial males secure breeding opportunities by defending groups of adult females from other males^3^. In contrast, in three-spined sticklebacks *Gasterosteus aculeatus*, rather than directly guarding females, territorial males defend spawning sites (nests), where they attract and mate with visiting females^4^. Territorial behavior also incurs costs, such as higher energy consumption, injury risk from competition and limited feeding opportunities^5,6^ so the development of this behavior will depend on the type and value of the resource being defended^5^. It will likely evolve when the benefits of controlling a territory outweigh the costs required to defend it.

In many animal taxa, large males adopt reproductive tactics such as territorial, bourgeois, and consort behavior to maximize their reproductive success, whereas smaller males adopt alternative tactics such as extra-pair mating, opportunistic mating, parasitic mating, sneaking, and satellite behavior^7,8^. These variations in mating behavior are referred to as alternative reproductive tactics (ARTs), in which individuals of the same sex use different tactics to achieve reproductive success^7,8^.

Loliginid squids display elaborate reproductive behaviors, including agonistic behavior (usually among males), courtship, mating, and post-copulatory mate guarding, which provide a unique model system to explore male ARTs^8,9^. In many species, males adopt ARTs such as consort and sneaker behaviors to increase their reproductive success^9^. Large consort males deposit their spermatophores near the opening of the oviduct inside the female’s mantle cavity using the “male-parallel” mating posture, in which the male clasps the female from underneath^8–11^. In contrast, smaller sneaker males use the “head-to-head” position, in which the male and female face each other directly, to place spermatophores near the female’s seminal receptacle, a sperm storage organ located on the buccal membrane. The females store sperm in these storage sites until spawning^8,9,12^. DNA fingerprinting has confirmed multiple paternities within both clutches and egg capsules in several species^11,13–16^ and shown that consort males generally achieve higher reproductive success than sneaker males^11,16^.

In addition to consort and sneaker males, solitary large males have also been observed around spawning grounds in several loliginids^11,17–19^. In Cape Hope squid *Loligo reynaudii*, solitary large males have been observed near egg masses in sandy spawning areas and reported to fight paired consort males to obtain access to females^18^. Similar behavior was documented in long-finned inshore squid *Doryteuthis* (*Amerigo*) *pealeii*^19^. However, no signs of territoriality were observed in these males in either species.

The spear squid *Heterololigo bleekeri* is a medium to large-sized, demersal species distributed in Japan, Korea, the East China Sea, and the Yellow Sea^20^. It is one of the commercially most important loliginids in Japan^16^ where it is caught widely in coastal waters around the main islands. The spawning season extends from winter to spring^21–23^ and eggs are laid in clusters attached to the undersurface of hard substrates such as rocks^20,22,24^. Fisheries often target mature, spawning squid^20,25^. Both consort and sneaker tactics have been reported in males^16^ with consort behavior observed in mature males with mantle lengths exceeding 221 mm^26^. The length of the spermatophore, shape of the sperm mass, length of the sperm flagellum and chemotaxis toward respiratory CO_2_ all markedly differ between sneaker and consort males, suggesting that the male reproductive tactics in this species are fixed^12,26,27^. Solitary males have also been reported in this species, and Wada (2005) documented these males as a “territorial type”^28^. However, quantitative measures of this behavior have not been reported.

To understand the behavior of solitary large males in loliginid squids requires focused observations for extended periods (several hours per day), but depths and diving conditions in the field can pose significant challenges for long-term observations^18^. A powerful alternative to field studies is to conduct observations in large experimental tanks. They can house many squid simultaneously, allowing extended observations on interactions between individuals near spawning substrates. In recent years, experiments in a large (300-ton) experimental tank have provided new insights into cephalopod behaviors that are difficult to observe in the field^29–31^.

To date, most studies of ARTs in *H. bleekeri* have been conducted either in confined tanks and net cages with many squid or tanks using only 2–3 squid^16,28,32^. In the present study, interactions between solitary males and other squid around spawning substrates were investigated in a large experimental tank using 15–17 squid. We describe their behavior, intra-sexual competition, and mating success, and report the first quantitative evidence of possible territorial behavior in male squid near a spawning substrate.

## Materials and methods

### Experimental design

**Fig. 1:**
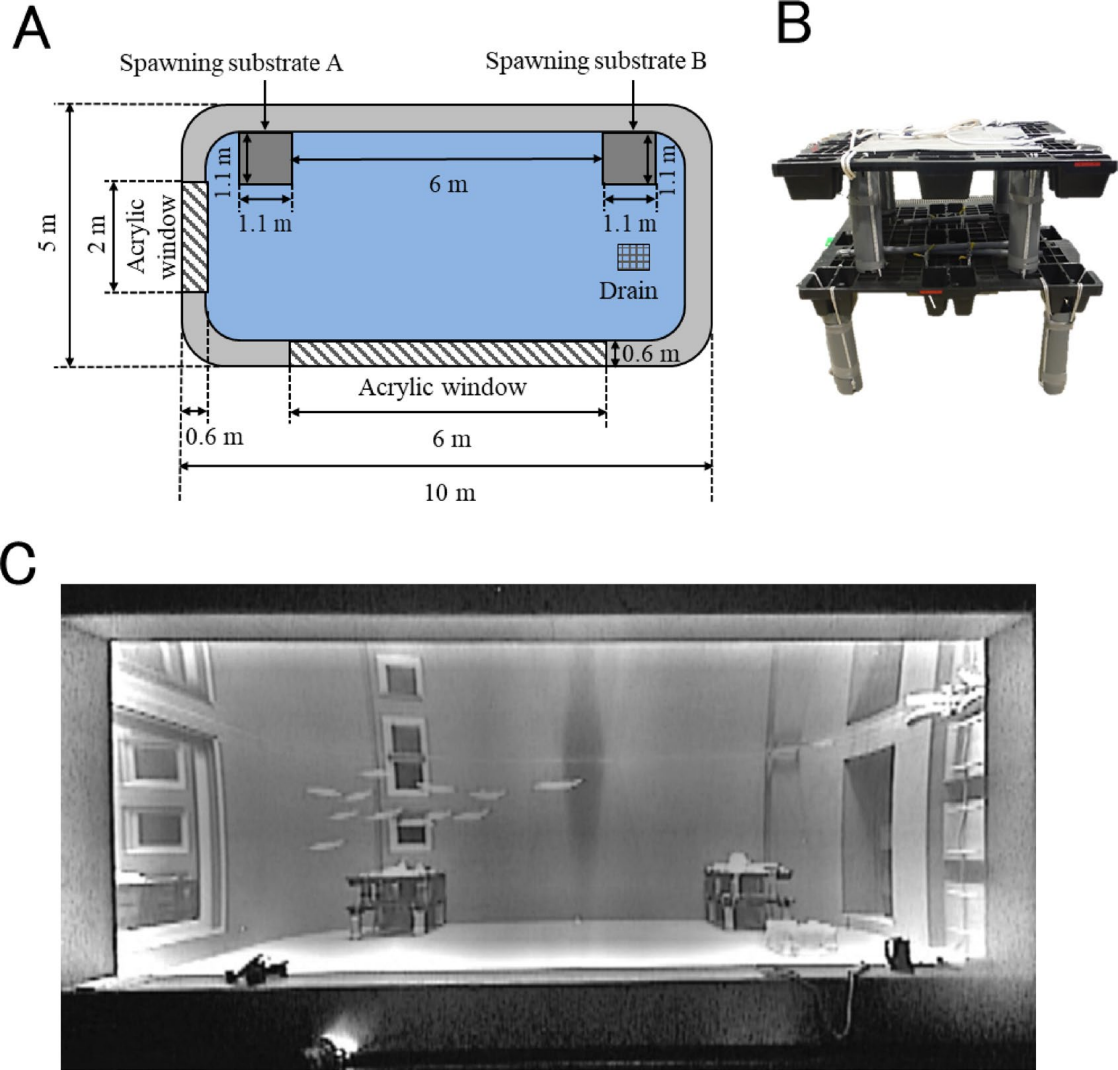
Experimental tank and spawning substrates. (**A**) View from above the tank. (**B**) An artificial spawning substrate. (C) Side view of tank showing squid and two spawning substrates.

Experiments were conducted in a 300 m^3^ tank (length 10 m, width 5 m, depth 6 m; Fig. 1 A) at the Hakodate Research Centre for Fisheries and Oceans, Hokkaido, Japan, during May 2–12, 2016. The tank contained filtered seawater to a depth of 3 m, and the water temperature was maintained at 12 °C using an overflow system with a low flow rate. The tank was lit using two LED lamps (each 400 W) above the tank pointed upwards. During the day (08:00–20:00), both lamps were used; at night (20:00–08:00), one was turned off.

The experiments were conducted using *H. bleekeri* collected in southern Hokkaido, Japan, using trap nets and jigs. Squid were sexed based on external morphology and measured (mantle length, ML). The reproductive organs of mature squid are easily observed without dissection, so external observations on the testes (white) and ovaries (yellow) were used to sex the squid. Internal examination of the reproductive organs following each experiment revealed that all squid were mature. To identify each male during the experiment, small ribbon tags were attached to the dorsal mantle. Care was taken to minimize the handling of the squid during the tagging. After tagging, each squid was kept briefly in a 1000-liter tank to confirm that the tags did not affect the swimming behavior.

The experiments comprised two trials (I and II) using identical rearing conditions. Trial I began with 12 females (mean ML ± standard deviation (SD), 205 ± 18.8 mm) and 5 males (M1 (286 mm), M2 (278 mm), M3 (272 mm), M4 (284 mm), and M5 (278 mm)) and continued for five days (127 h). Trial II began with 11 females (198 ± 19.2 mm) and 4 males (M6 (314 mm), M7 (288 mm), M8 (244 mm), M9 (224 mm)) and continued for three days (73 h). Different males were used in the two trials. For females, 7 of the 12 individuals in Trial I were reused in Trial II to minimize the number of animals used in the experiment. The purpose of the study was to observe the behavior of large males near spawning substrates, so small sneaker males were not included. The squid were fed liberally once a day with Pacific saury *Cololabis saira* fillets, and squid that died were removed using a brail net. Trial I was terminated when the number of males reached one. Trial II was terminated when the tank was no longer available for use due to an unrelated experiment by another research team.

Ethical approval was not required from Hokkaido University (affiliation of the first author during the study) to conduct this study because cephalopods are not covered by the university’s animal experiment regulations. Nevertheless, animal welfare was carefully considered. All experiments adhered to the recommendations for reporting animal research in the ARRIVE guidelines^33^. All rearing procedures were conducted in accordance with the “*Guidelines for the Care and Welfare of Cephalopods in Research*”^34^ and followed the experimental guidelines set by Hokkaido University.

Before each trial, two spawning substrates (A and B) comprising plastic panels and pipes (1.1 m × 1.1 m × 1.1 m; Fig. 1B) were placed at the bottom of the tank 6 m apart (Fig. 1 A, B and C) to test whether male behavior differed depending on the presence or absence of egg capsules on the substrates.

### Behavioral observations

Observations were conducted through two acrylic viewing windows on the tank’s side (length 6 m, height 2.2 m) and end (length 2 m, height 2.2 m). Before each trial, the squid were observed for at least three hours to ensure that none displayed weakened or abnormal swimming behavior.

### Proximity of squid to spawning substrates

During each trial, in-person observations were conducted daily for one hour every six hours (i.e., four times per day). At five-minute intervals during each in-person observation period, the squid nearest to each spawning substrate was recorded. These observations through the two viewing windows enabled us to accurately identify the squid closest to the spawning substrates at the optimal angle, even when multiple squid were present near the substrates. If a male, it was identified based on its ribbon tag, and if a female, it was recorded simply as “female”. In cases when all squids were observed near one of the spawning substrates, recording the outermost individual of the school as the closest squid to the other substrate could be misleading when determining if the squid were associated with that substrate. Therefore, when no squid was present in the half of the tank containing the substrate, the closest individual was recorded as “none”. Differences in the number of times each male was recorded as the closest squid were tested using the χ^2^ test for each spawning substrate. When the result of the χ^2^ test was significant and the number of males recorded as the nearest squid was more than three, multiple comparisons were conducted using the exact binomial test for each comparison pair after Bonferroni correction. To ensure the reliability of statistical conclusions, the χ^2^ test was not performed when any category had an expected count less than 5. During Trial I, three males died before the end of the trial. Thus, the analysis was conducted for each period when the number of males in the tank was same.

### Agonistic behavior

During each in-person observation period, we recorded the number of times each male displayed agonistic behaviors, such as “chase” (i.e., active pursuit of a male by another male) and “rush and grab” (a highly escalated competitive behavior in which a male rushes towards another with arms tightly closed, then opens and throws them outward, or directly grabs the male)^35^. Differences in the number of agonistic behaviors among males were tested using the χ^2^ test. Where the result of the χ^2^ test was significant and the number of males that displayed agonistic behavior was more than three, multiple comparisons were conducted using exact binomial tests for each comparison pair after Bonferroni correction. In Trial I, the analysis was conducted for each period when the number of males in the tank was same.

### Mating

During the in-person observations, the number of matings, mating position (male-parallel or head-to-head), and mating duration (the time from the start of the male-parallel or head-to-head posture to when the pair separated) was recorded for each male. After a mating was observed, the female was closely observed to determine if it spawned.

During the periods when in-person observations were not conducted, mating behavior was recorded using a video recorder (Sony HDR-CX590V Handycam, Sony, Minato, Tokyo, Japan). In Trial I, it was set at the side of the tank and recorded activity in the half of the tank with Substrate B. In Trial II, it was set at the side of the tank initially and recorded activity in the half of the tank with Substrate A. After 38 h from the start, it was set at the end of the tank and recorded activity in the entire tank. Three hours in Trial I (70–72 h and 90–91 h after the start) and five hours in Trial II (32–37 h after the start) were excluded from the analysis because the camera was tilted and did not record properly.

### Egg capsules

At the start of each in-person observation period, the substrates were examined using binoculars to determine if any egg capsules were attached, and the time when an egg capsule was first observed was recorded for each spawning substrate. At the end of each trial, the spawning substrates were retrieved, and the numbers of attached egg capsules on each were counted.

## Results

### Proximity of squid to spawning substrates and agonistic behavior

***Trial I***. In Trial I, a total of 22 one-hour, in-person, observation periods were conducted, and the squid nearest to the two substrates was recorded 286 times. The trial began with five males and lasted 127 h; M3 died 91–96 h after the start of the trial, M2 died after 109–114 h, and M1 died after 115–120 h.

During the first observation period (0–1 h), all squid formed a single school, with M4 occasionally approaching Substrate B, but soon returning to the school. In all subsequent observations (6–127 h), a male usually remained near Substrate B (M1 during 0–115 h, M4 during 120–127 h, Supplementary Fig. S1), while the other squid schooled in the center or opposite half of the tank (see Supplementary Video 1 online).

**Fig. 2:**
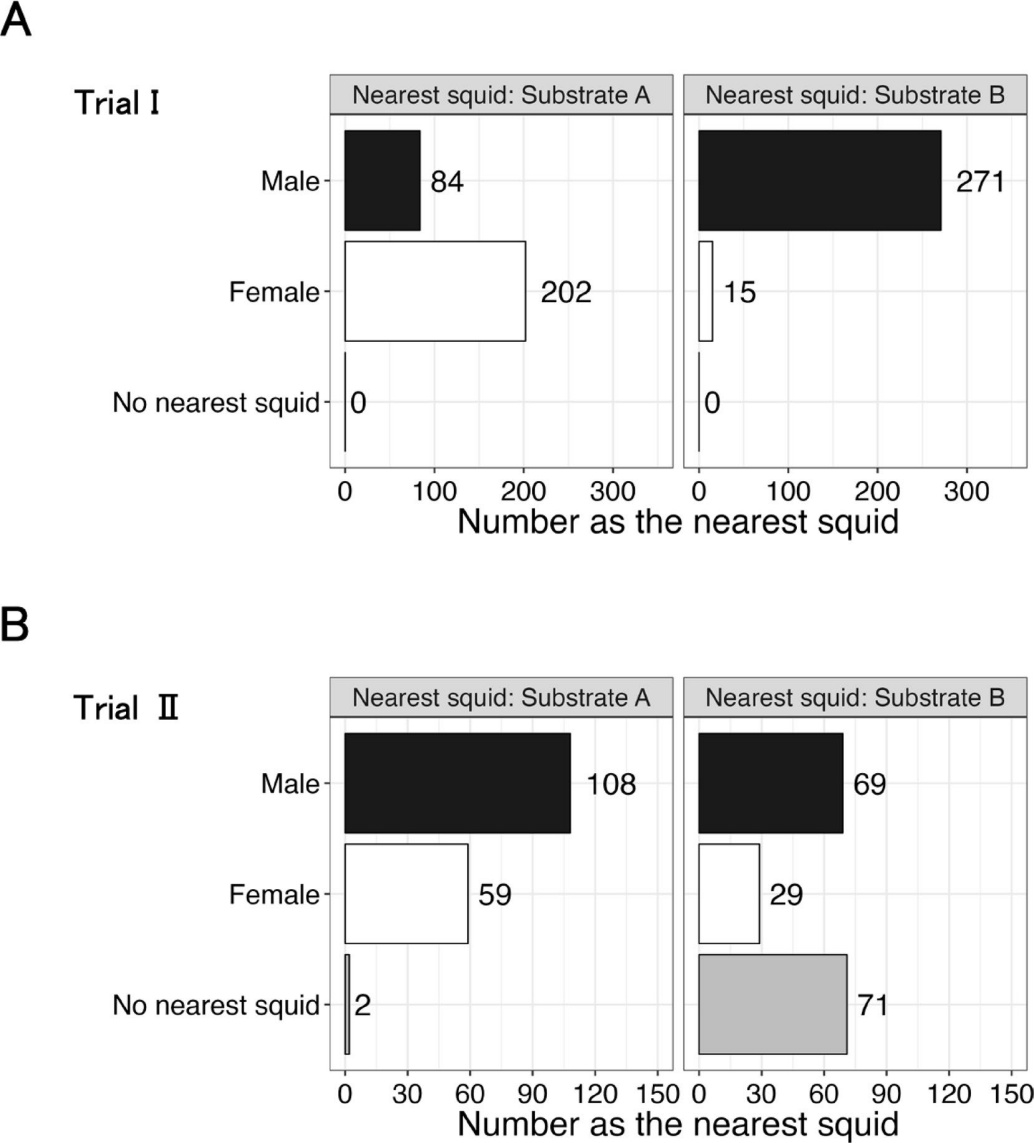
The number of times males and females were recorded as the nearest squid to Substrate A and Substrate B in both trials. “No nearest squid” indicates that no squid was present in the half of the tank containing the substrate. (**A**) The number recorded in Trial I (**B**) The number recorded in trial II.

**Fig. 3:**
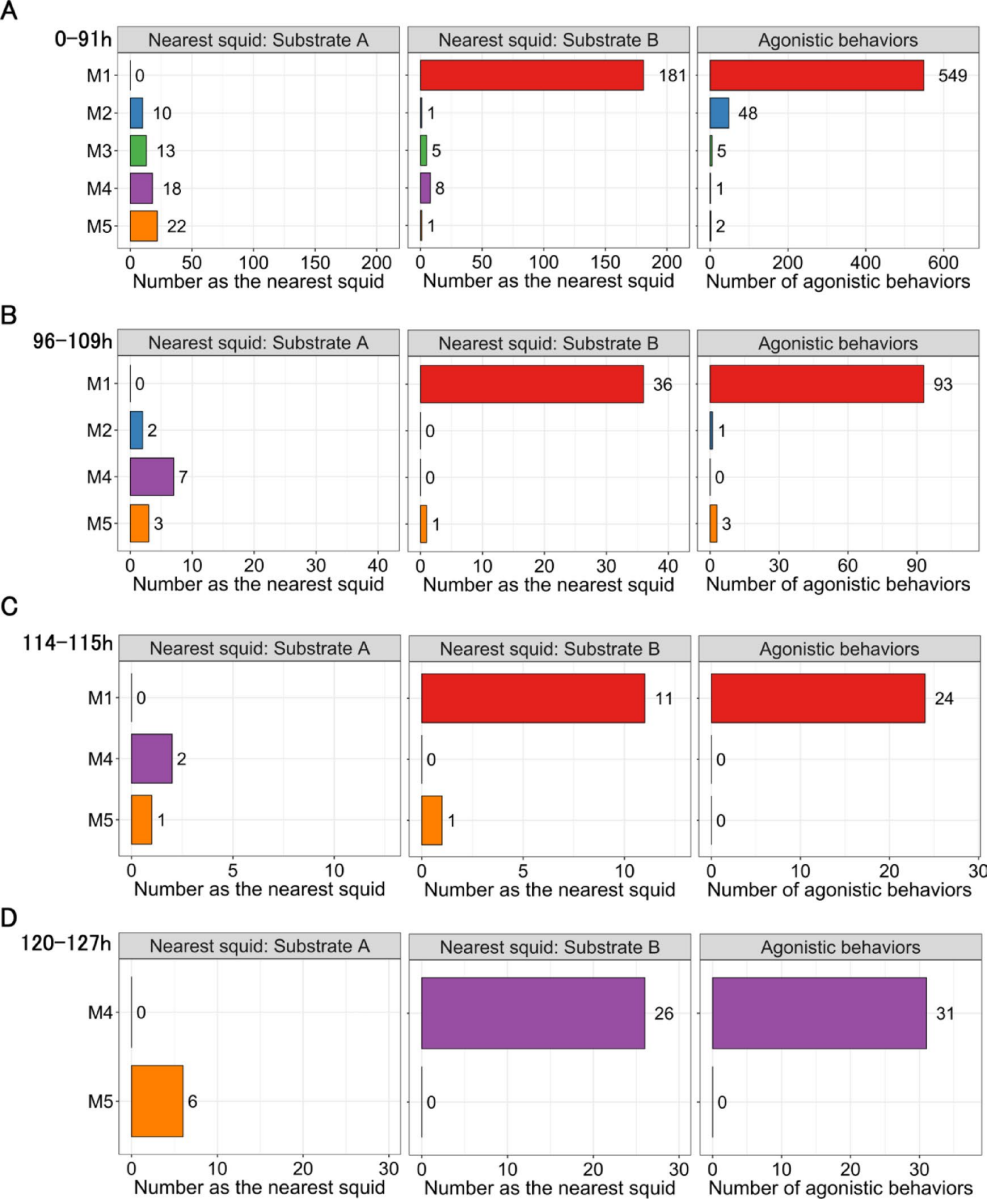
The number of times each male (M1–M5) was recorded as the nearest squid to Substrate A (left column) and Substrate B (middle column), and the number of agonistic behaviors displayed by each male (right column) during visual observations in Trial I. (**A**) When all five males were alive. (**B**) When four males remained (following death of M3). (**C**) When three males remained (following death of M2). (**D**) When two males remained (following death of M1).

At Substrate B, the squid nearest to the substrate was a male in 95% of the observations (Fig. 2); in 84% of those observations, the nearest squid was M1 (Fig. 3). While alive (0–115 h), M1 was recorded as the closest squid more often than any other male and swam as the solitary male. When 4–5 males were present (0–109 h), it was the nearest squid significantly more often than the other males (Fig. 3A–B, *p* < 0.01, exact binomial tests). When three males were present (114–115 h), M1 was still the nearest squid, but statistical comparison was not conducted due to the small sample size (Fig. 3 C). After it died, it was replaced as the nearest squid by M4 (Fig. 3D).

At Substrate A, no individual dominated as the nearest squid (Fig. 3). When all five males were alive (0–91 h), four (M2, M3, M4, and M5) were each recorded as the nearest squid (Fig. 3 A), but none was recorded significantly more often than the other three. M1 was never recorded as the nearest male as it swam alone around Substrate B. Limited data prevented analyses when there were fewer than four males.

In terms of agonistic behavior, 757 male agonistic bouts were observed in the 22 in-person observation periods. Most bouts were initiated by solitary males (M1 during 0–115 h, M4 during 120–127 h) near a substrate when approached by another male from a school. When M1 was alive (0–115 h), it initiated 92% of the agonistic bouts (Fig. 3A–C), which was significantly more often than in other males (*p* < 0.01, exact binomial tests). When only two males (M4 and M5) remained (120–127 h), M4 replaced M1 as the nearest male to Substrate B and initiated all agonistic bouts when approached by M5 (Fig. 3D). Rush and grab behavior, an indicator of active exclusion, was observed only seven times in Trial I, six of which were performed by solitary males toward other males (5 times by M1, once by M4, Supplementary Fig. S3).

***Trial II***. In Trial II, a total of 13 one-hour, in-person, observation periods were conducted, and the squid nearest to the two substrates was recorded 169 times. The trial began with four males and lasted 73 h; all males survived until the end of the trial. During the first five periods (0–25 h), the squid formed a single school, but during the sixth period (30–31 h), males began to approach the substrates (Supplementary Fig. S2).

**Fig. 4:**
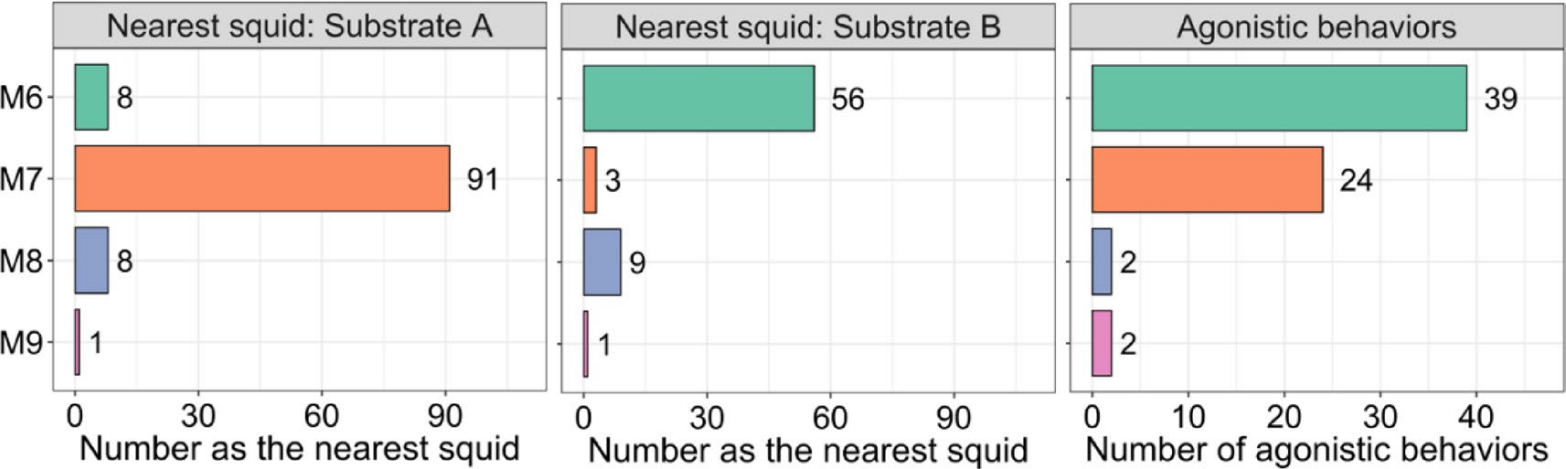
The number of times each male (M6–M9) was recorded as the nearest squid to Substrate **A** (left column) and Substrate **B** (middle column), and the number of agonistic behaviors displayed by each male (right column) during visual observations in Trial II.

At Substrate A, when a nearest squid was recorded, 65% of the time it was a male (Fig. 2); in 84% of those observations, the nearest squid was M7 (Fig. 4), which displayed solitary behavior near Substrate A. It approached the substrate in the sixth observation period (30–31 h) and remained the nearest squid significantly more often than any other male (Fig. 4, *p* < 0.01, exact binomial test).

At Substrate B, when a nearest squid was recorded, 70% of the time it was a male (Fig. 2). M6 was the nearest squid in 81% of the observations that males were recorded as the nearest squid (Fig. 4), which displayed solitary behavior around Substrate B. This male was recorded as the nearest squid significantly more often than any other male (Fig. 4, *p* < 0.01, exact binomial tests).

During the final 31 h of the trial, M6 and M7 remained near the spawning substrates as the other squid schooled (Supplementary Fig. S2).

In terms of agonistic behavior, 67 male agonistic bouts were observed in the 13 in-person observation periods. Bouts were initiated significantly more often by M6 and M7, each acting as a solitary male near one of the two substrates, than by the other two males (Fig. 4, *p* < 0.01, exact binomial tests) and were most often initiated by M6 near Substrate B towards M7 in the center of the tank. Rush and grab behavior was observed only seven times in Trial II, six of which were performed by solitary males toward other males (2 times by M6, 4 times by M7, Supplementary Fig. S4).

### Mating and spawning

Mating was observed 37 times in person and in the video recordings; all were in the male-parallel position. Before mating, when a female left a school and approached the spawning substrate, it inspected the substrate by touching the undersurface of the substrate or the egg capsules, and was usually accompanied by either a solitary male near the substrate or one or more males from the school. This was often followed by agonistic behavior between males and mating. If the female mated with a male from the school, the male was usually threatened by a solitary male near the substrate and returned to the school alone after mating. If the female mated with a solitary male near the substrate, the male usually guarded the female after they mated (and in some cases, during spawning). Subsequently the females usually returned to the school alone.

**Fig. 5:**
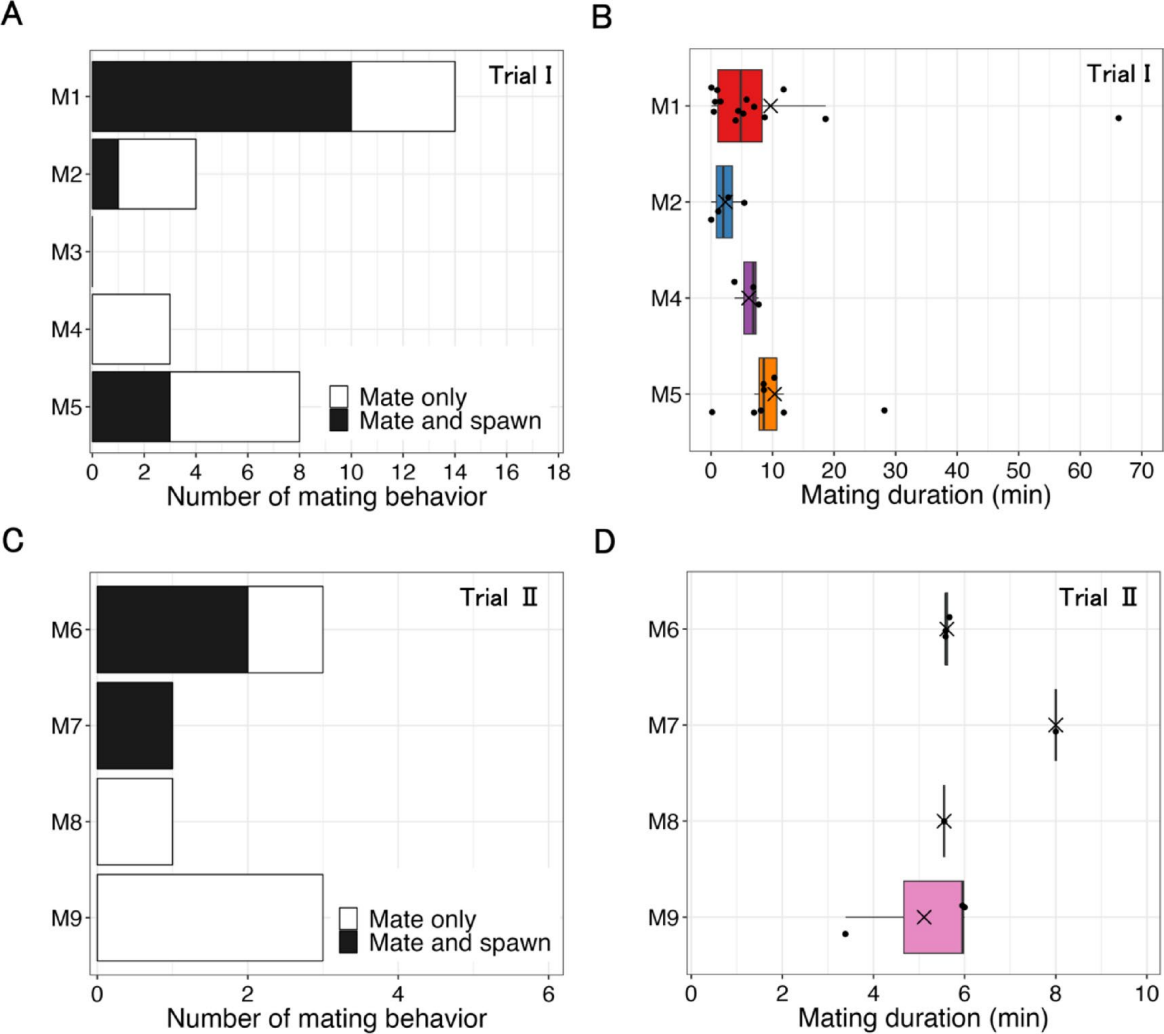
Mating behavior recorded in Trial I and Trial II. (**A**) Number of mating behaviors for each male squid in Trial I. (**B**) Box-and-whisker plots of mating duration for each male squid in Trial I. (**C**) Number of mating behaviors for each male squid in Trial II. (**D**) Box-and-whisker plots of mating duration for each male squid in Trial II. Note: in (**B**) and (**D**), the thick line in each box plot is the median. The cross is the average. The limits of the boxes indicate the lower (Q1) and upper quartile (Q3) of the distribution. The length of the box is the interquartile range (IQR). The limits of the whiskers are the data points within the calculated minimum value (Q1–1.5 × IQR) and the calculated maximum value (Q3 + 1.5 × IQR). The data points outside the whiskers are outliers.

***Trial I***. In Trial I, mating was recorded 29 times (Fig. 5 A). All mating occurred when 4–5 males were present. All males mated except M3. The solitary male (M1) mated the most often (Fig. 5 A), but the frequency did not differ significantly among the four males that mated (M1 vs. M2 *p* = 0.19, M1 vs. M4 *p* = 0.08, M1 vs. M5 *p* = 0.08, M2 vs. M4 *p* = 1.00, M2 vs. M5 *p* = 1.00, M4 vs. M5 *p* = 1.00, exact binomial tests). Mating lasted from 2 to 3975 s (approximately 1.1 h) (Fig. 5B, median = 347, mean ± SD = 509.6 ± 21.4), and the mating duration did not differ significantly among the males (*p* = 0.11, Kruskal-Wallis test).The solitary male (M1) mated 14 times: 8 started inside Substrate B when females entered, and 4 began nearby (Supplementary Table S1). The remaining two started farther away but moved to the substrate during mating, which resulted in spawning. In contrast, other males (M2, M4, M5) stayed with the group and usually mated away from the substrate. Of their 15 matings, only 5 began inside Substrate B (2 by M2, 1 by M4, 2 by M5), typically after the male followed a female into the substrate, sometimes while being attacked by M1.

Spawning was observed during or following 14 of the 29 matings (Fig. 5 A). All spawning occurred on Substrate B. Spawning immediately followed mating most often when females mated with M1, but statistical comparisons were not performed due to the small sample size. A female squid spawned immediately after being introduced into the tank, so egg capsules were present on Substrate B at the onset of the first in-person observation. At the end of the Trial I, 288 egg capsules were attached to Substrate B, whereas no egg capsules were found on Substrate A.

***Trial II***. In Trial II, mating was recorded eight times (Fig. 5 C). All males mated at least once. Due to the small sample size, it was not possible to statistically compare the frequency of mating by each male. Mating lasted 203–480 s (Fig. 5D, median = 337.5, mean ± SD = 342.9 ± 6.8), and the mating duration did not significantly differ among the males (*p* = 0.49, Kruskal-Wallis test).

Spawning was observed following three of the matings (twice following matings with M6, and once following mating with M7). The solitary male M6, which remained near Substrate B, initiated two of the three matings near Substrate B (Supplementary Table S2). The remaining matings were initiated inside Substrate B when the female entered the substrate. A single mating was observed for the solitary male M7, which remained near Substrate A. This mating was initiated near Substrate A and led to spawning. Other males (M8 and M9) initiated matings outside the substrates, but these matings did not result in spawning.

On Substrate A, egg capsules were first observed at the start of the first in-person observation period. On Substrate B, egg capsules were first observed 42 h after the start of the trial. At the end of the trial, there were 116 egg capsules attached to Substrate A and 106 attached to Substrate B.

## Discussion

In this study, individually identified squid were observed over longer periods (127 h in Trial I, 73 h in Trial II) than in previous studies (2–63 h per trial^16^ 2–5 h per trial^32^. In addition, squid were provided more space (5.9–10.0 m^3^ per individual) than in previous studies (4.3 m^3^ per individual^16^ 0.9 m^3^ per individual^32^ 2.1 m^3^ per individual^28^. The tank used in this study allowed for observations on squid behavior under more field-like conditions.

Observations on the swimming behavior of *H. bleekeri* males near the two substrates and their agonistic interactions suggest some formed territories near the substrates. Individual males (M1 and M4 in Trial I, M6 and M7 in Trial II) were observed to remain near the spawning substrates and display more frequent agonistic behavior than other males. Although the number of observations was limited, rush and grab behavior was more frequently performed by solitary males toward other males, which suggests that territorial males not only show more agonistic behavior, but also actively exclude other males. The substrates in this study could be considered a reproductive resource, so the male behavior fulfills the conceptual definition of territoriality (i.e., having a fixed space from which an individual actively excludes competitors from a resource)^1^. Territorial behavior associated with reproduction has been reported in males in many animal taxa^2,5,36^ but to our knowledge, this is the first report of such behavior in a squid using quantitative data.

The benefits of territorial tactics in squid are possibly related to their complex fertilization dynamics. For territoriality to be favored by natural selection, the benefit gained from maintaining a territory must outweigh the energetic cost of defending it^6^. Territorial males of *H. bleekeri* did not gain exclusive access to females for mating or mate longer than non-territorial males, but by establishing territories, they might increase their mating opportunities with females when the chances of paternity are high. In *L. reynaudii*, the last males to mate with a female prior to spawning are thought to sire most of the spawned eggs^11,18^. In *D. pealeii*, male reproductive success has been shown to be influenced by the interval between mating and spawning^37^. Sperm precedence is not yet well understood in *H. bleekeri*, but in controlled mating trials, the last males to mate with females before spawning fertilized 87–100% of the eggs, suggesting that last male sperm precedence occurs in this species^9,16^ While our sample size was small, in both trials, territorial males tended to be the last male to mate before spawning. Territoriality in squid may differ from that observed in other taxa, as the benefit is not the monopolization of females or mating opportunities themselves, but rather the monopolization of mating opportunities at times optimal for fertilization.

Another possible reason for establishing territories is related to where *H. bleekeri* spawns. Unlike many loliginid species that attach their egg capsules to gravel or sandy seafloor and form dense spawning aggregations in open spawning beds^9,11,20,38^* H. bleekeri* females attach their egg capsules to the undersurface of small and spatially heterogeneous hard substrates such as rocks, which limit the number of spawning pairs that can use an area^9,20,24^ and might encourage the formation of territories. This limitation may lead to localized competition among males for access to these scarce reproductive resources, and the spatial restriction of the resource can create conditions under which territory defense yields reproductive benefits that outweigh the energetic costs. The absence of territorial behavior in other squid species that utilize sandy spawning substrates^19^ may be explained by the relatively uniform and widespread distribution of such substrates, where the energetic costs of territory defense may exceed the potential reproductive benefits. The territorial behavior observed in *H. bleekeri* in this study indicates that the spatial distribution and structural characteristics of reproductive resources can be a critical determinant of reproductive tactics in squid. If this hypothesis holds true, it is plausible that other squid species utilizing similarly structured spawning substrates may also exhibit territorial behavior. For example, the European squid *Loligo vulgaris* deposits egg masses on hard, ceiling-like surfaces, similar to those used by *H. bleekeri.* Although territorial behavior in *L. vulgaris* has not been reported, repeated sightings of the same male at a spawning site over several consecutive days have been reported^39^. This suggests the possible formation of male reproductive territories associated with specific spawning substrates, similar to the pattern observed in *H. bleekeri*. Other unique features of *H. bleekeri* include that it mates in the male-parallel position for much longer than other loliginids and is the only species in which males place spermatophores inside the female oviduct rather than inside the mantle cavity near the oviduct, which is common in other loliginids^9,12^. To better understand the benefits gained from establishing territories, further studies combining behavioral observations and paternity analysis are needed.

Detailed quantitative data focusing on the process of how squid territories are formed were not available in this experiment. However, some of the behavioral descriptions and the formation of territories only on substrates with egg capsules suggest that the presence of egg capsules might influence the process of male territory formation. The two spawning substrates were identical in size, shape and material, but in both trials, territorial behavior was observed only on substrates where egg capsules had previously been attached (Substrate B in Trial I, and both substrates in Trial II). Arnold (1962) reported that captive *D. pealeii* females spawn when eggs are placed in their tank and suggested the eggs act as a visual stimulus that elicits spawning in other females^40^. Since then, egg capsules have also been shown to trigger increased competition between males in this species during the breeding season^41–43^. The effect of egg capsules on the behavior of *H. bleekeri* males is not known, but the presence of eggs might also trigger the males to establish territories on substrates that are expected to attract mature females. Squid territoriality may be caused by the presence of egg capsules in the spawning substrate, which stimulates males to change their behavior from schooling to solitary and agonistic behavior. Future experiments manipulating the presence/absence, number, and sensory cues of the egg capsules would be useful to verify these predictions.

In this study, the largest males in both trials were the ones that exhibited territoriality and initiated most of the agonistic bouts. These results suggest that larger males have a competitive advantage over rivals when establishing territories. Although territorial behavior has not been observed in *Doryteuthis plei*, larger males in this species are known to be more likely to win contests for temporary access to a female^44^. In several animal taxa (e.g., insects^45^ fishes^4^ reptiles^46^, larger males have been found to have an advantage in establishing reproductive territories. However, the relationship between body size and territoriality is not always straightforward, and factors such as body condition, color patterns and pheromones also play important roles in male territoriality^47–49^. The relationship between male body size and territorial behavior in squid should be carefully tested in future investigations, taking the above factors into account.

In conclusion, the results from our extended observations suggest that *H. bleekeri* males form territories around spawning substrates. This is the first report to confirm the occurrence of territoriality in squids using individual quantitative data, including the frequency of closest access to spawning substrate and the frequency of competition among males. Territories were formed only on substrates where egg capsules were present, and territorial males remained alone on the spawning substrate while continuing to exclude other males. Territorial males did not monopolize the females themselves or mating opportunities, suggesting that the benefits of territorial tactics are related to the complex fertilization patterns of this species. We proposed a novel reproductive tactic in squids and several specific hypotheses that can be tested in the future. Further studies focusing on male territoriality in loliginid squids are awaited to better understand their elaborate male sexual selection.

## Supplementary Information

Below is the link to the electronic supplementary material.

Supplementary Material 1

Supplementary Material 2

## Data Availability

All data generated or analyzed during this study are included in this published article and its Supplementary Information Files.
