# Supplementary material for: First quantitative evidence of territorial behavior in squid near spawning substrates
Source: Sci Rep. 2025 Aug 8;15:29061. doi: 10.1038/s41598-025-14308-1 (PMC12334606; doi:10.1038/s41598-025-14308-1)
Supplement: Supplementary file 2 — Supplementary Material 2 [file 41598_2025_14308_MOESM2_ESM.docx]

**Supplementary information**

First quantitative evidence of territorial behavior in squid near spawning substrates

**
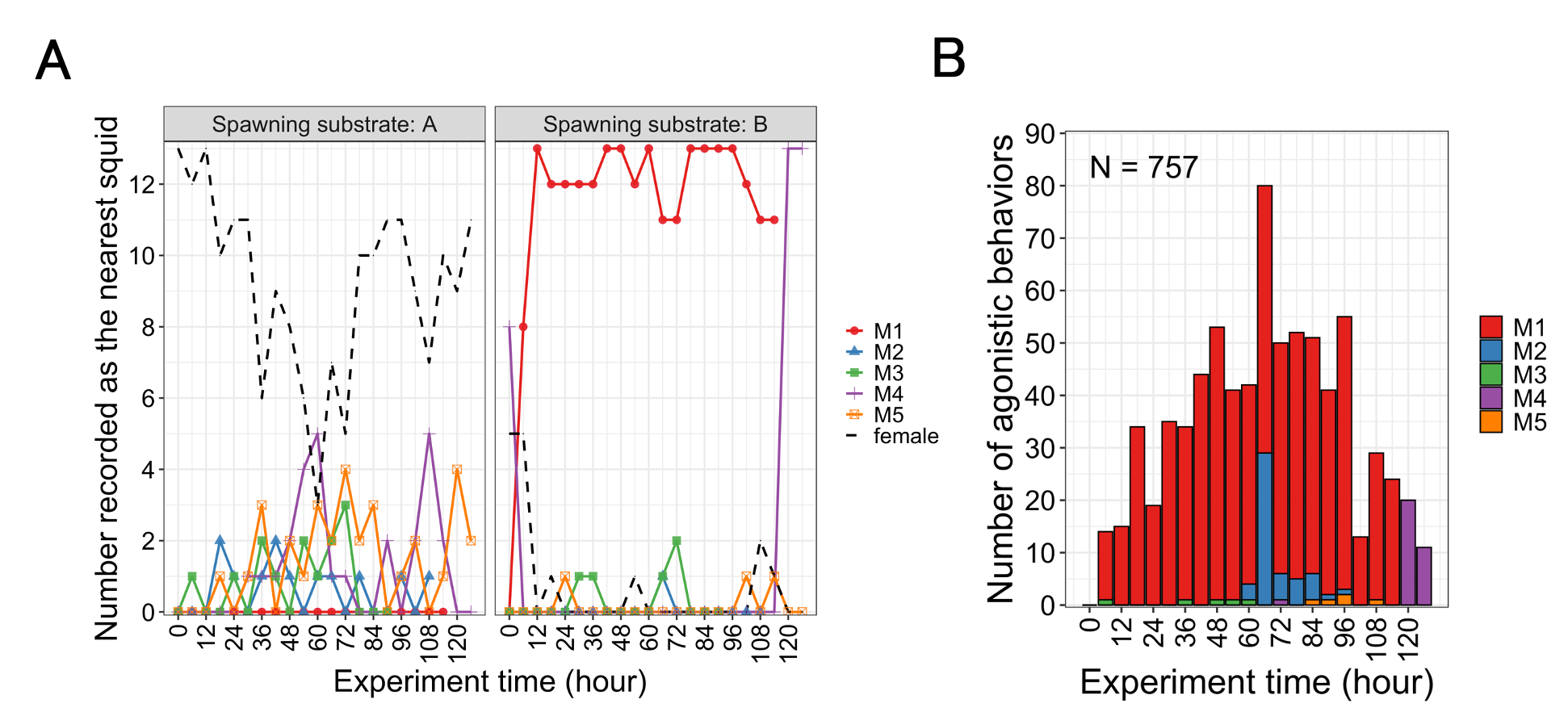
**

**Figure S1.** Observation results from Trial I. (A) The number of times each squid was recorded as the nearest squid to the substrates. (B) The number of agonistic behaviors initiated by each male squid.

**
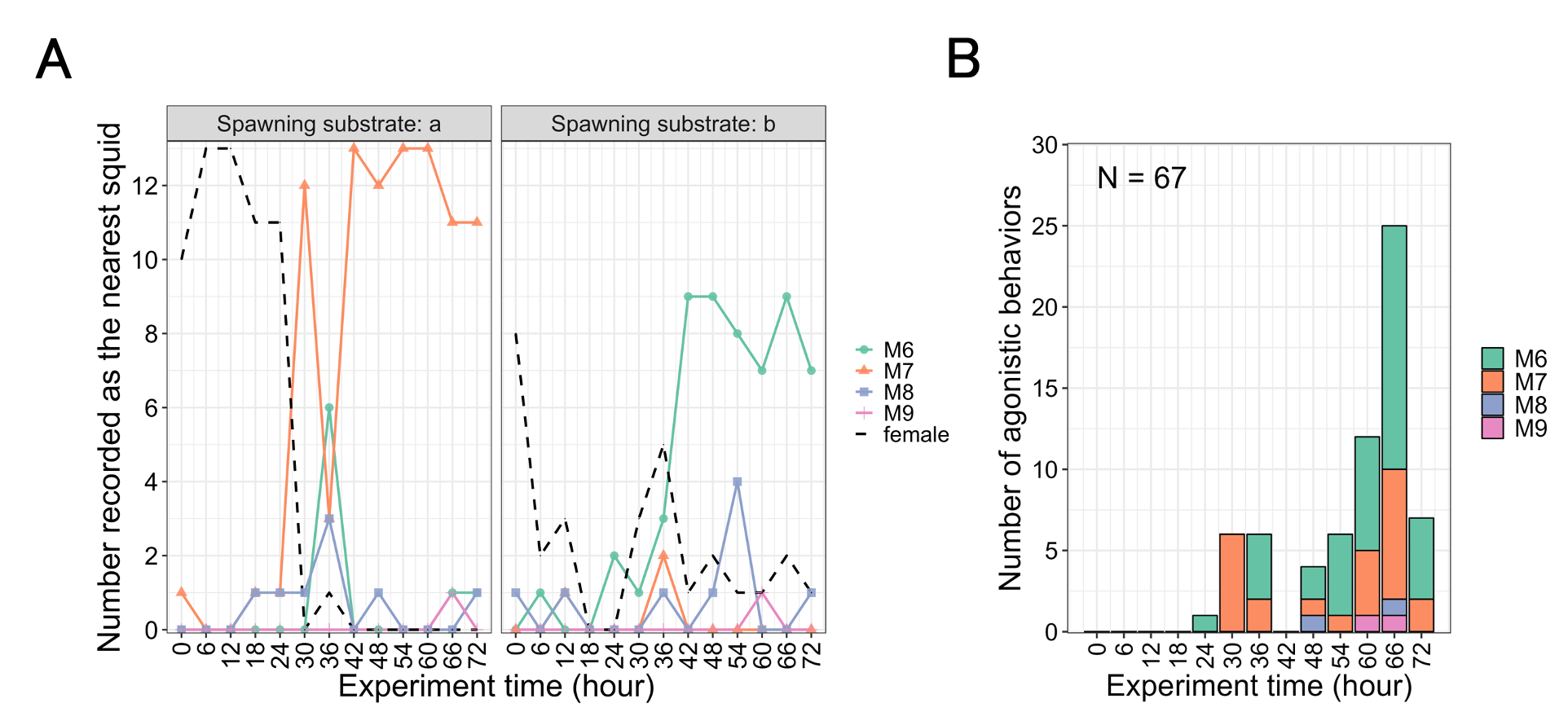
**

**Figure S2.** Observation results from Trial II. (A) The number of times each squid was recorded as the nearest squid to the substrates. (B) The number of agonistic behaviors initiated by each male squid.


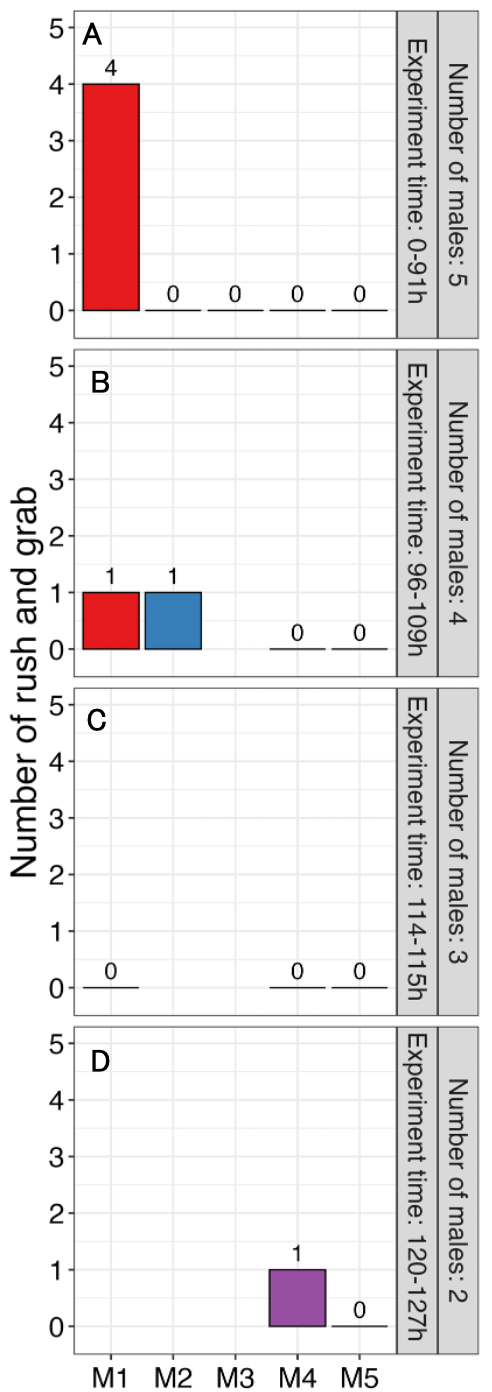


**Figure S3.**　﻿ The number of times rush and grab behavior was displayed by each male during visual observations in Trial I. (A) When all five males were alive. (B) When four males remained (following death of M3). (C) When three males remained (following death of M2). (D) When two males remained (following death of M1).


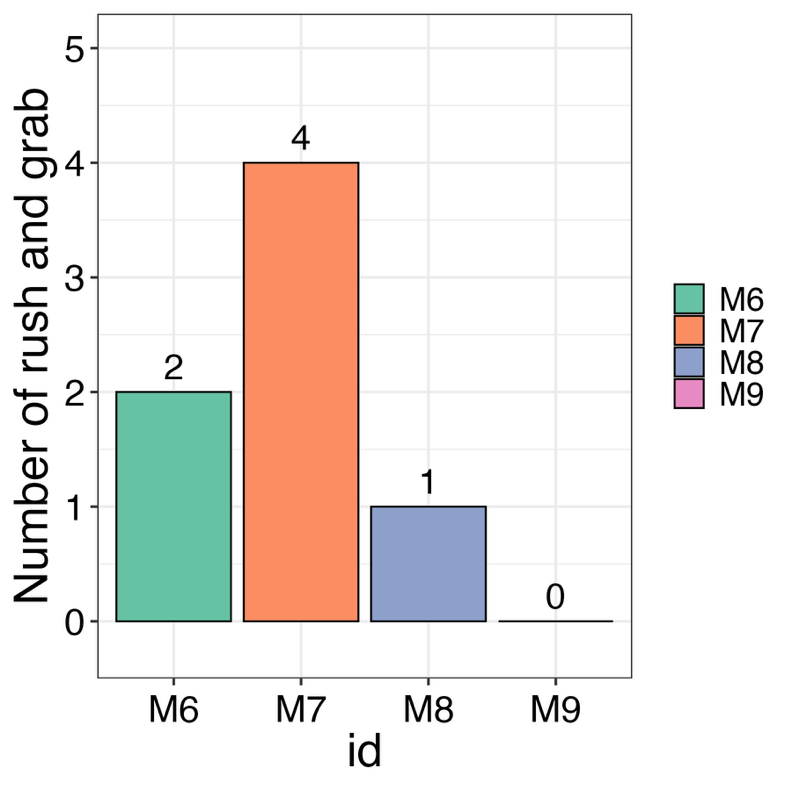


**Figure S4.**　The number of times rush and grab behavior was displayed by each male during visual observations in Trial II.

**
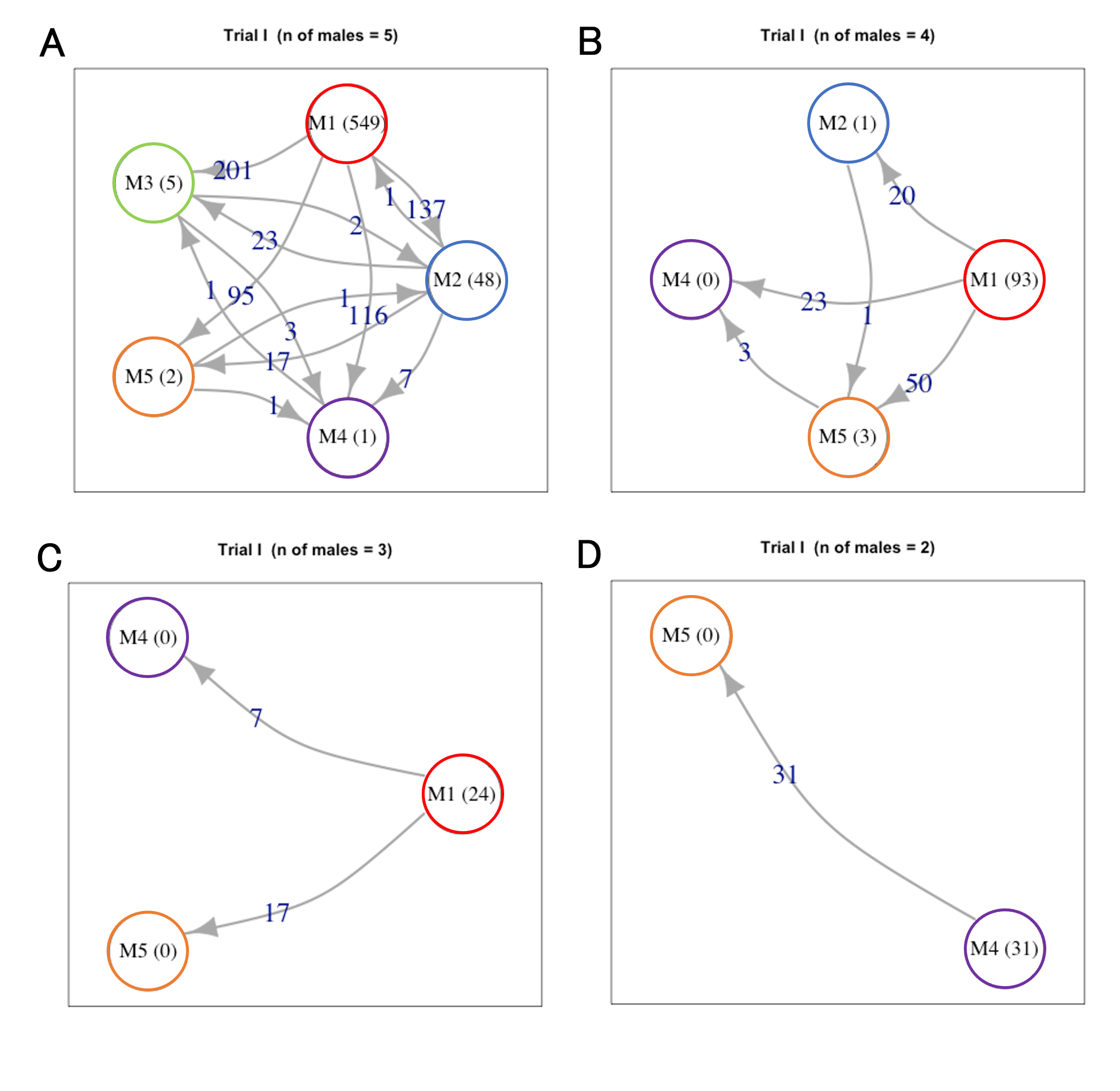
**

**Figure S5.** Summary of agonistic behaviors displayed in visual observations during Trial I as the number of males decreased from 5 (A) to 2 (D). Numbers in parentheses indicate total numbers of agonistic behaviors initiated by the male. Arrows point toward targets of agonistic behavior, and the numbers on the arrows indicate the number of agonistic behaviors.

**
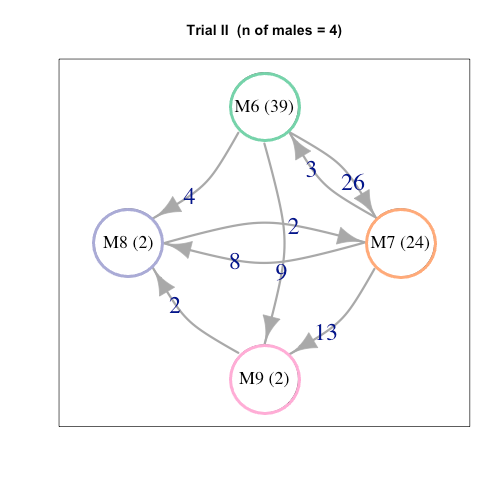
**

**Figure S6.**　Summary of agonistic behaviors displayed in visual observations during Trial II. Numbers in parentheses indicate total numbers of agonistic behaviors initiated by the male. Arrows point toward targets of agonistic behavior, and the numbers on the arrows indicate the number of agonistic behaviors.

**﻿ Table S1.**　﻿ Mating behavior recorded in Trial I. MP = male‐parallel position. Number and letter in parentheses indicate the mating duration in seconds and the spawning substrate (A or B) to which the mating pair approached or entered, respectively. Bold font indicates that spawning was observed during or immediately after the mating. Shaded times indicate nighttime observations. Dashes indicate that M3 had already died and was no longer in the tank. Area A: half of tank with Substrate A. Area B: half of tank with Substrate B.

**Table S2.**　﻿ Mating behavior recorded in Trial Ⅱ. MP = male‐parallel position. Number and letter in parentheses indicate the mating duration in seconds and the spawning substrate (A or B) to which the mating pair approached or entered, respectively. Bold font indicates that spawning was observed during or immediately after the mating. Dashes indicate that M3 had already died and was no longer in the tank. Area A: half of tank with Substrate A. Area B: half of tank with Substrate B.

**Table S3.**　﻿The number of times each male was recorded as the nearest squid in visual observation of Trial I. Statistical analysis was conducted for each period when the number of males in the tank was the same. “Not conducted” indicates that there was no statistical analysis due to small sample size. Different letters in parentheses within the same row indicate significant differences in multiple comparisons using exact binomial tests. Dashes indicate that the squid had died and been removed from the tank.

**Table S4.**　﻿ The number of agonistic behaviors displayed by each male in visual observation of Trial I. Statistical analysis was conducted for each period when the number of males in the tank was the same. Different letters in parentheses within the same row indicate significant differences in multiple comparisons using exact binomial tests. Dashes indicate that the squid had died and been removed from the tank.

**Table S5.**　﻿ The number of times each male was recorded as the nearest squid in visual observation of Trial II. Different letters in parentheses within the same row indicate significant differences in multiple comparisons using exact binomial tests.

**Table S6.**　﻿ The number of agonistic behaviors displayed by each male in visual observation of Trial Ⅱ. Different letters in parentheses within the same row indicate significant differences in multiple comparisons using exact binomial tests.

**Supplementary Video 1.**　﻿ Territorial behavior of a male *Heterololigo bleekeri* near the spawning substrate. This video presents a side view of the large experimental tank during Trial I, in which M1 remains near the spawning substrate and frequently exhibits agonistic bouts toward other males.
